# “Ageism” Is Associated With Self-Reported Multidimensional Frailty in Community-Dwelling Older Subjects: A Population-Based Study

**DOI:** 10.3389/fmed.2021.734636

**Published:** 2022-02-18

**Authors:** Sabrina Zora, Alberto Cella, Stefano Poli, Nicola Veronese, Elena Zini, Paola Giannoni, Valeria Pandolfini, Claudio Torrigiani, Alberto Pilotto

**Affiliations:** ^1^Geriatrics Unit, Department of Geriatric Care, Orthogeriatrics and Rehabilitation, E.O. Ospedali Galliera, Genova, Italy; ^2^Department of Education, University of Genoa, Genova, Italy; ^3^Department of Geriatrics, University of Palermo, Palermo, Italy; ^4^Department of Interdisciplinary Medicine, University of Bari Aldo Moro, Bari, Italy

**Keywords:** perceived age discrimination, self-assessed frailty, multidimensional prognostic index, older, epidemiology

## Abstract

Ageism is a stereotyping, prejudice and discrimination against people, based on age. Ageism may impact the quality of life and the care of older people, a problem that can be greater when the older person is “frail.” However, few studies explored the role of frailty as a factor related to ageism. The aim of this study was to assess the association between perceived age discrimination (PAD), i.e., ageism, and multidimensional frailty in a cohort of community-dwelling older adults. We enrolled 1,337 community-dwelling subjects over-65 years that filled out a structured questionnaire to collect psycho-socio-economic and behavioral information. Multidimensional frailty was assessed by the SELFY-Multidimensional Prognostic Index Short-Form (SELFY-MPI-SF). PAD, over the past 5 years, was assessed based on explicit criteria. Overall, 83 out of 1,337 participants (6.2%) reported PAD. These subjects were older, more frequently women, with greater economic difficulties, lower level of cultural fruition, social network and psychological well-being, and a greater degree of frailty compared to their counterparts. After adjustment for age and gender, multidimensional frailty (SELFY-MPI-SF score) and negative affectivity were the two only “predictors” significantly associated with PAD (SELFY -MPI-SF, Odds Ratio: 1.19, 95%CI: 1.029–1.370; PANAS negative: Odds Ratio: 1.06, 95%CI: 1.033–1.099). In conclusion, self-reported frailty and negative affectivity are independently associated with PAD in community-dwelling older people. Interventions to prevent and treat frailty could be useful to reduce ageism and improve the well-being of the older people.

## Introduction

Ageism is a complex phenomenon that encompasses stereotypes, prejudice and discrimination against older adults, old age and aging (1). Age discrimination is becoming increasingly important in a time of rapid population aging and there is a growing literature on ageism focusing on the negative effects of age discrimination both at the individual and societal level (2, 3). According to World Health Organization (4), ageism is the most frequent social form of discrimination and COVID-19 perpetuated and triggered several forms of public ageism (5, 6).

A recent review reported that most of the studies explored other-directed ageism and only a small number investigated self-directed ageism and subjective perceived ageism (7).

Indeed, it has been reported that subjective perceived age discrimination (PAD) produces detrimental effects (8) with harmful consequences for physical, mental, and social health (9, 10). Research has generally focused on PAD in everyday situations (9, 11, 12). Different studies showed that it was associated with older age (12–14), gender (15), years of education (12, 13, 16, 17), lower socioeconomic status (12, 13, 16, 17), ethnicity (13), not being married (17–19), being unemployed (16, 19) and depressive symptoms (20). Ageism is also related to social victimization (21) as well as elder abuse (22).

Frailty is an age-related clinical condition characterized by a decline in physiological capacity across several systems, that increase the vulnerability to stressors resulting in an increased risk of negative outcomes, such as hospitalization, falls, institutionalization and mortality (23). Frailty is a multidimensional condition, with physical and psychosocial factors playing an important role in its development (24). Indeed, frailty can be adequately captured by the comprehensive geriatric assessment (CGA) (25), by exploring several domains such as physical and cognitive function, nutrition, multimorbidity, polypharmacy and social determinants that are key factors in the assessment and management of frailty in older people (26). The Multidimensional Prognostic Index (MPI) is a prognostic tool based on a standard CGA that showed a good discrimination and accuracy for mortality and other negative outcomes both in hospital (27) and community settings (28). Nowadays, the MPI has becoming one of the most used frailty instruments in older adults (29). Recently, a self-administered version of the MPI (SELFY-MPI) (30) and its short-form version (SELFY-MPI-SF) (31) were developed and validated in community-dwelling older people both showing strong agreement and precision when compared with the standard version of the MPI based on the CGA.

Ageism and frailty are conditions that share the possibility of being associated with negative outcomes for the older people; however, very few studies included frailty as a factor potentially related to ageism (32, 33) and little is known of the possible relationship between these two conditions. Recently, increased attention was given to ageism in healthcare setting since it is widely recognized that behavioral and attitudinal approach toward older adults requires more awareness among health-care professionals (34).

The aim of this study was to investigate the association between frailty and PAD in a community-dwelling older population.

## Materials and Methods

### Design and Participants

The present study is part of the PRESTIGE project (Involved and Resilient: Aging in Genoa), aimed to prevent frailty and social vulnerability in community-dwelling older residents in the metropolitan area of Genoa, Italy. The survey was carried out between October 2019 and February 2020 in accordance with the World Medical Association's 2008 Declaration of Helsinki, the guidelines for Good Clinical Practice, and the Strengthening the Reporting of Observational Studies in Epidemiology (STROBE) guidelines (35).

The inclusion criteria were: (1) age 65 years or over; (2) community-dwelling people who attended the University of the Third Age (U3A– an international movement whose aim is encouraging the education of retired members of the community) in Genoa according to a lifelong learning program for subjects in their “third age” of life; (3) absence of acute clinical conditions; (4) ability to provide informed consent.

The Ethical Committee of Department of Education of the University of Genoa (DISFOR), Genoa, Italy approved the present study on 5 September 2019; study number 030. All participants read and signed the informed consent form and all participants' records, and personal information were rendered anonymous before statistical analysis.

### Measures

All study participants filled out a socio-demographics questionnaire including age, gender, and marital status. In addition, further information was collected on:

- *Education*, assessed according to the International Standard Classification of Education (ISCED: 0 = no qualification; 1 = primary school; 2 = secondary school; 3 = vocational school of 2–3 years; 4 = high school; 5 = bachelor's degree; 6 = PhD); data was then re-coded into a three-point categorical scale (lower/average/higher);
- *Economic difficulties*, ascertained by evaluating the purchasing power of the subjects' family. Participants were asked whether they had difficulties in dealing with ordinary expenses (three-point scale: none/average/above average);
- *Social engagement*, assessed by mean of the Lubben Social Network Scale (36), a 12-item self-reported scale to measure social engagement including family and friends. The total score is calculated through the sum of all items. The score ranges between 0 and 60: a higher score reflects a stronger social engagement;
- *Cultural fruition*, assessed through a three-point scale (lower/average/higher), according to a previously published method (37), that explores leisure activities such as reading books, magazines and newspaper, going to the theater or cinema, participating in cultural events, hobbies, using media, traveling abroad and attending public places;
- *Positive and Negative Affect Scale*, assessed by means of the Positive and Negative Affect Scale (PANAS) (38), a 20-item questionnaire with 10 items measuring positive affect (e.g., excited, inspired) and 10 items measuring negative affect (e.g., upset, afraid). Each item is rated on a five-point Likert Scale, ranging from 1 = Very Slightly or Not at all to 5 = Extremely, to measure the extent to which the affect has been experienced during the past weeks;
- *Self-reported victimization*, assessed though three specific questions that explored whether participants had been victims of robbery, fraud, physical assaults over the last 5 years (the possible answer was on a dichotomic choice yes/not).

### Frailty

The Self-Administered MPI Short Form (SELFY-MPI-SF) (31) was used to assess frailty, by combining information on the following eight domains through eight self-administered scales:

(a) *Functional status* was evaluated by the Barthel Activities of Daily Living (ADL) sub-scale (39) which measures the level of dependence/independence in six daily personal care activities such as feeding, bathing, personal hygiene, dressing, fecal and urinary continence and toilet use. This scale can be self-assessed (40);

(b) *Mobility* was assessed through the Barthel mobility sub-scale (39), which assess the following three abilities: getting in and out of bed/chair, walking and going up and down the stairs. This scale can also be self-administered (40, 41).

(c) *Independence in the instrumental activities of daily living* (IADL) was assessed through the self-administered version of Lawton's IADL scale (42) that explores the independence in eight activities such as telephone use, grocery shopping, meal preparation, housekeeping, laundry, travel, medication, handling finances.

(d) *Cognitive status* was investigated using the self-administered cognitive screening test Test Your Memory (TYM) (43), a 10-task performance test that explores different cognitive domains: orientation, ability to copy a sentence, semantic knowledge, calculation, verbal fluency, similarities, naming, visuo-spatial abilities and recall of a previously copied sentence. The score ranges between 0 and 50, with higher scores indicating better cognitive function (43).

(e) *Nutritional status* was measured with the self-administered version of the Mini Nutritional Assessment Short-Form (MNA-SF) (44), that includes information on anthropometric measures (body mass index and weight loss), decline in food intake, mobility, recent psychological stress and neuropsychological problems;

(f) The *number of drugs* regularly used by the participant;

(g) *Comorbidity* was assessed by investigating the number of pathologies, among the first 13 categories of the Cumulative Illness Rating Scale (CIRS) (45), which require chronic drug therapy;

(h) *Co-habitation status* was evaluated by exploring three options: living alone, in an institution or with family members.

For each domain, a tripartite hierarchy is adopted based on conventional cut-off points: a score of 0 indicates no problems, 0.5 minor problems and 1.0 major problems. The sum of all these eight domains was divided by eight to obtain the SELFY-MPI-SF score (31), whose value is between 0 and 1 (the higher the score, the greater the degree of frailty).

### Perceived Age Discrimination

PAD was ascertained explicitly by asking participants if they have ever been discriminated against for their age over the last 5 years; the possible answer was on a dichotomic choice “yes” or “no” (46).

### Statistical Analysis

Main descriptive statistics were reported as absolute and relative (%) frequencies for categorical variables or as means with their standard deviation (SD) or median and interquartile range (IQR), depending on the normality of the distribution.

To compare the subjects' characteristics at baseline, “*p”* values were calculated by using Student's *t*-test or Mann–Whitney U test, where appropriated, for independent samples for continuous variables, and chi-square test for categorical ones.

The association between PAD (main dependent variable) and selected covariates was tested by means of a binary logistic regression analysis model. The predictors included in the final model were all the variables that reached a *p* < 0.10 in the univariate analysis. A backward model was applied to obtain the best set of factors associated with PAD. Odds ratios (ORs) and 95% confidence intervals (CIs) were used to compare the prevalence of associated factors by PAD status.

All analyses were performed by means of the SPSS 25.0 for Windows (SPSS Inc., Chicago, Illinois). All statistical tests were two-tailed and statistical significance was assumed for a p <0.05.

## Results

The study sample included a total of 1,337 community-dwelling older people aged 65 years or over. with a mean age of 77.4 years (SD: 7.4, range 65–107 years). Women were 56.0% and almost a third of the subjects lived alone (*n* = 429; 31.5%).

Eighty-three participants (6.2%) declared to have been discriminated against for their old age (PAD-group) during the 5 years before the interview. Table 1 shows the characteristics of the study participants, grouped according to the presence or absence of PAD: the subjects included in the PAD-group were older (*p* = 0.03), more frequently women (*p* = 0.046), with greater economic difficulties (*p* = 0.001), lower level of cultural fruition (*p* = 0.022), social network (on average about two points less on the Lubben scale, *p* < 0.001), higher prevalence of negative affectivity (on average about 3.5 points more on the PANAS-negative scale, *p* < 0.001), and lower prevalence of positive affectivity (about two points less on the PANAS-positive scale, *p* = 0.032) compared to participants who did not declared PAD.

**Table 1:** Participants' characteristics.

	**Overall study sample**		**Perceived Age Discrimination**		
			**No**	**Yes**	
Number of subjects (%)		1,337 (100)	1,254 (93.8)	83 (6.2)	
**Variable**					***p***
**Age, years**	Mean (SD)	77.46 (7.43)	77.35 (7.38)	79.14 (8.04)	0.033
	Min/max	65–107	65–101	65–107	
**Gender**, ***n*** **(%)**					
	Male	592 (44.3)	564 (45.0)	28 (33.7)	0.046
	Female	745 (55,7)	690 (55.0)	55 (66.3)	
**Level of education (ISCED recoded)**, ***n*** **(%)**					
	Lower	760 (56.8)	706 (56.3)	54 (65.1)	0.146
	Average	416 (31.1)	392 (31.3)	24 (28.9)	
	Higher	161 (12.0)	156 (12.4)	5 (6.0)	
**Housing status**, ***n*** **(%)**					
	At home, with relatives/ caregivers	901 (67.4)	850 (67.8)	51 (61.4)	0.058
	Residential facility	15 (1.1)	12 (1.0)	3 (3.6)	
	At home, alone	421 (31.5)	392 (31.3)	29 (34.9)	
**Economic difficulties**, ***n*** **(%)**					
	None	838 (62.7)	801 (63.9)	37 (44.6)	0.001
	Average	256 (19.1)	235 (18.7)	21 (25.3)	
	Above average	243 (18.2)	218 (17.4)	25 (30.1)	
**Level of cultural fruition**, ***n*** **(%)**					
	Lower	490 (36.6)	448 (35.7)	42 (50.6)	0.022
	Average	256 (19.1)	245 (19.5)	11 (13.3)	
	Higher	591 (44.2)	561 (44.7)	30 (36.1)	
**Self-reported victimization**, ***n*** **(%)**					
	no	1,031 (77.1)	969 (77.3)	62 (74.7)	0.336
	yes	306 (22.9)	285 (22.7)	21 (25.3)	
**Lubben Social Network Scale**					
	Median (IQR)	15.4 (11.7–19.4)	15.7 (11.9–19.5)	12.9 (8.5–18.3)	<0.001
**PANAS - positive**					
	Median (IQR)	32 (25–38)	32 (25–38)	29 (21–36)	0.028
**PANAS - negative**					
	Median (IQR)	16 (12–21)	16 (12–21)	19 (14–26)	<0.001
**SELFY-MPI-SF**					
	Median (IQR)	0.19 (0.06–0.31)	0.19 (0.06–0.25)	0.25 (0.06–0.88)	<0.001

*ISCED, International Standard Classification of Education; PANAS, Positive and Negative Affect Scale; SELFY-MPI-SF, Multidimensional Prognostic Index-Short Form*.

**Figure 1:**
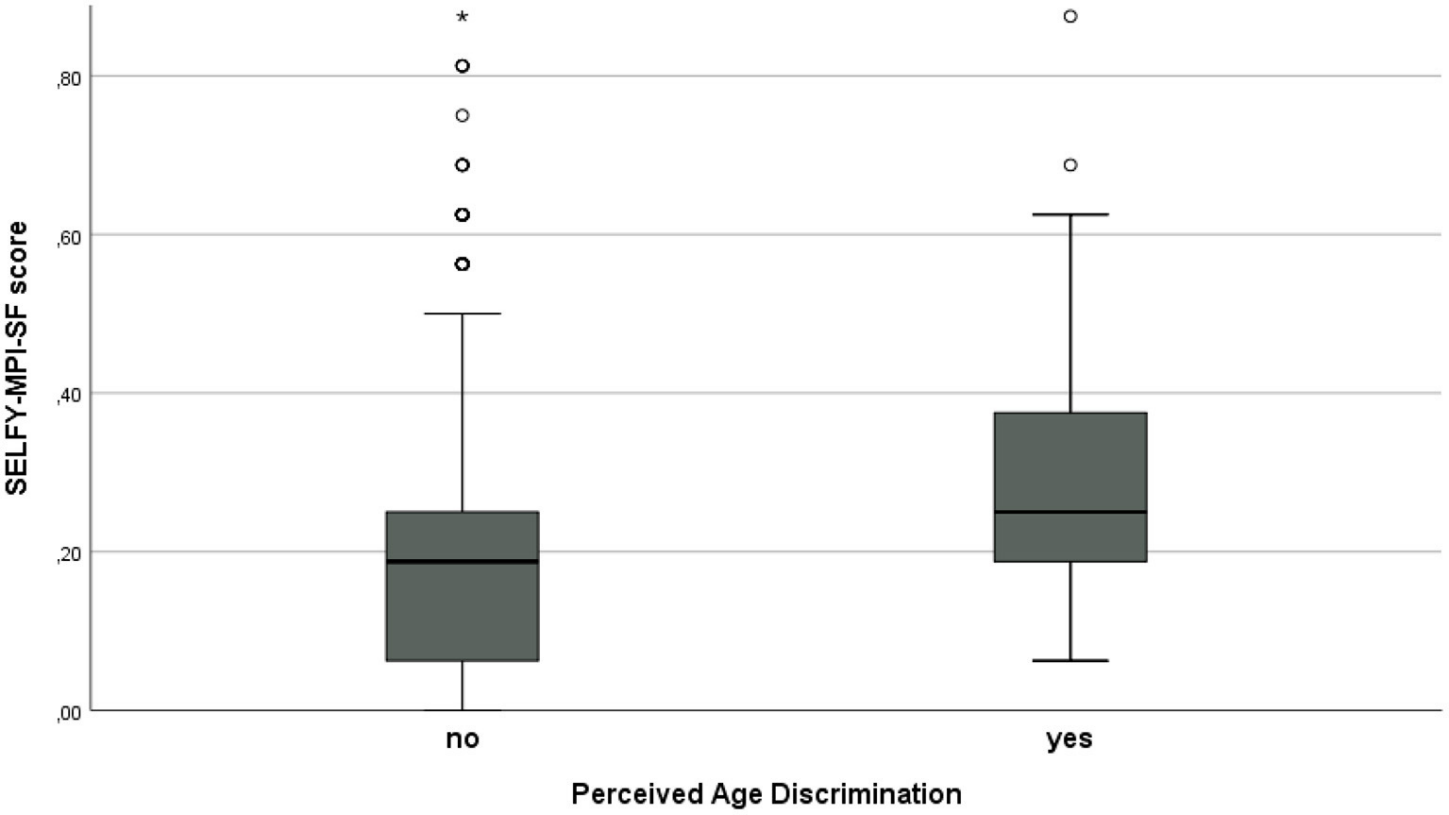
Box plot of SELFY-MPI-SF score in participants reporting or not PAD (*p* < 0.001). Data are reported as medians with their interquartile ranges and outliers, by the presence or not of perceived age discrimination. ^*^p < 0.05.

**Figure 2:**
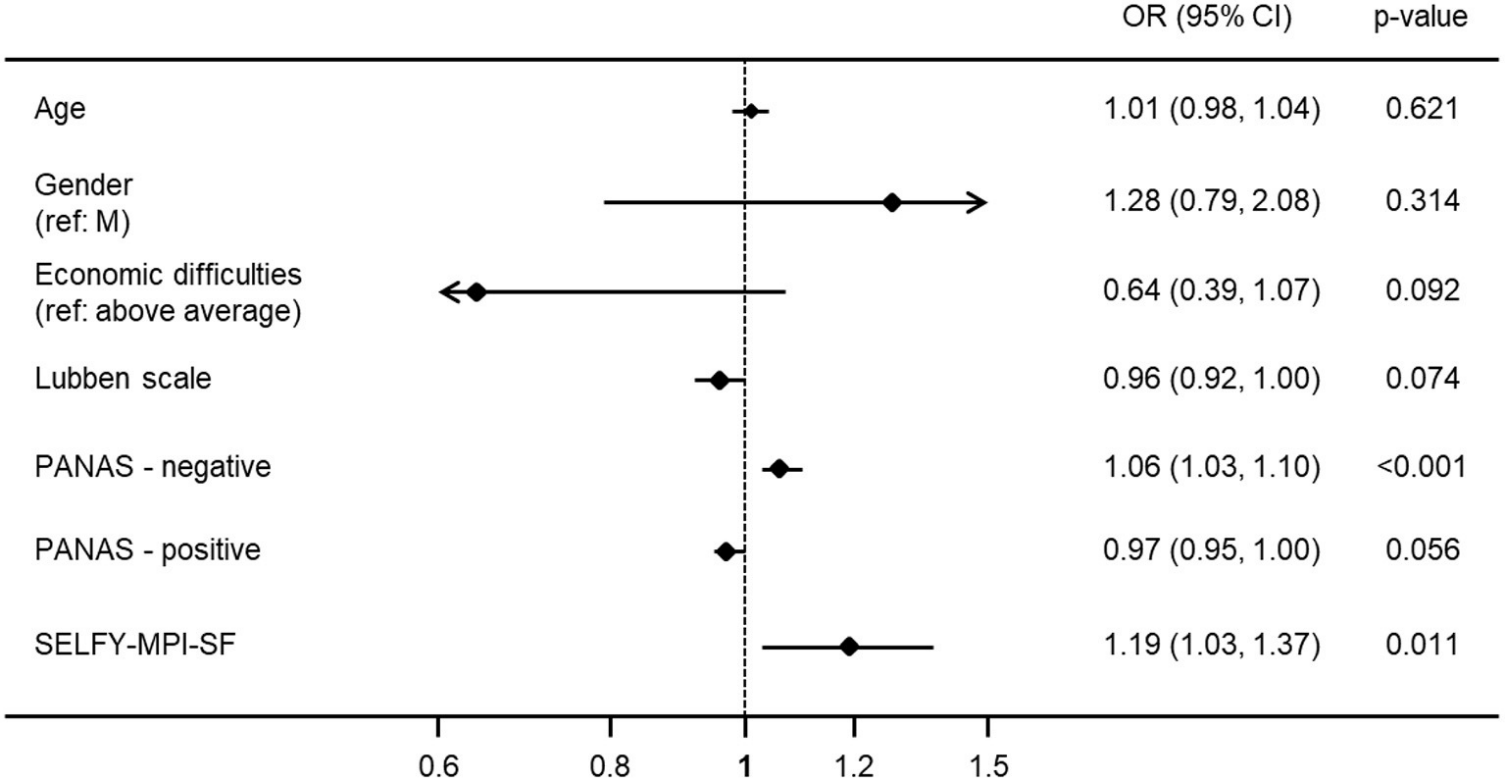
Predictors of PAD (Forest plot). Variables entered in the binary logistic regression analysis: age, gender, economic difficulties, Lubben Social Network Scale, Level of cultural fruition, PANAS-negative, PANAS-positive, SELFY-MPI-SF (see methods). In this analysis, the SELFY-MPI-SF score was multiplied by 10 and the variable “Economic difficulties” was dichotomized in “none/average” vs “above average”.

As shown in Table 1; Figure 1, a significantly higher level of frailty was observed in participants included in the PAD group than in subjects who did not report PAD: median SELFY-MPI-SF score = 0.25 (IQR 0.06–0.88) vs. 0.19 (IQR 0.06–0.25), respectively (*p* < 0.001). After adjustment for age and gender, the logistic binary regression analysis demonstrated a significant association between PAD and the SELFY-MPI-SF score (OR 1.19, 95%CI 1.03–1.37) and the PANAS-negative scale (OR 1.06, 95%CI 1.03–1.10), thus showing that only the degree of frailty and the extent of negative affect were independent predictors of PAD (Figure 2).

## Discussion

In this study we explored the association between ageism and self-assessed frailty in a large sample of community-dwelling older people. A relatively small proportion of the study participants enrolled in the PRESTIGE project experienced PAD (6.2%), a percentage lower than that reported in other studies (12, 13, 47, 48). This finding can be explained by the characteristics of the participants included in the study, i.e., socially and culturally active older people with a low degree of physical and cognitive limitations. Nevertheless, this study contributes to provide relevant information on the relative role of frailty and several socio-economic, cultural, and psychological factors in fostering the subjective perception of age-based discrimination in older people.

Most studies address people's attitudes toward older people, but few explores the subjective PAD of older subjects themselves (7, 46). In our study, a greater degree of frailty and a poor psychological well-being constitute the personal characteristics with the strongest association with subjective perceived ageism. While the association with depressive symptoms had already been stressed in other studies (20), the association of PAD with frailty is a new finding not previously highlighted. One study (32) investigated the cross-sectional association of ageist attitudes with frailty in Veterans 50 years and older but neither explicit nor implicit ageist attitudes were found associated with frailty. In our sample each increase of one decimal point in the SELFY-MPI-SF value is associated with about 20% increase in the likelihood of feeling a victim of ageism.

It should be also emphasized that the extent of the association of frailty and negative affects with PAD attenuate in our survey the role of other factors that emerged in previous studies (12–15), such as age, gender, cultural factors, and economic conditions.

The present study has some limitations. First, the study sample including active seniors attending U3A can be considered mostly a “low risk” group according to the MPI risk of frailty categories (26, 27). Therefore, a selection bias in our findings is possible. Second, the choice of ascertaining the presence of subjective ageism with an explicit question that only admitted “yes” or “no” as an answer, may not have caught more hidden forms of ageism. Third, multidimensional frailty was assessed only through a self-reported tool: even if SELFY-MPI has a good agreement with standard MPI, some differences could be present using a standard form of MPI. Finally, this study does not allow to define the mechanisms of association between frailty and PAD.

As researchers, we are interested in potential interventions against ageism and the first step is to develop appropriate strategies and to target interventions on specific more vulnerable older populations. For this reason, further studies are needed to verify whether interventions aimed at preventing or treating frailty are also capable of reducing PAD.

## Conclusion

Perceived age discrimination (PAD) in community-dwelling older people is associated with the degree of individual frailty, target of possible interventions aimed both at reducing ageism and improving the well-being of the older adults.

## Data Availability Statement

The raw data supporting the conclusions of this article will be made available by the authors, without undue reservation.

## Ethics Statement

The studies involving human participants were reviewed and approved by Comitato Etico della Liguria. The patients/participants provided their written informed consent to participate in this study.

## Author Contributions

AP planned the study, supervised the data analysis, and the paper submission. SP planned the study, contributed to revising the paper, and wrote the section Design and Participants. AC performed all statistical analysis and wrote the remaining sections of the paper. NV supervised all statistical analysis and contributed to revising the paper. SZ wrote the remaining sections of the paper. VP helped to plan the study and wrote the section Statistical Analysis. CT planned the study and wrote the section Frailty. EZ and PG collected data. All authors contributed to the article and approved the submitted version.

## Funding

This work was supported by Fondazione CARIGE (Stronger, less frail GRANT 2018).

## Conflict of Interest

The authors declare that the research was conducted in the absence of any commercial or financial relationships that could be construed as a potential conflict of interest.

## Publisher's Note

All claims expressed in this article are solely those of the authors and do not necessarily represent those of their affiliated organizations, or those of the publisher, the editors and the reviewers. Any product that may be evaluated in this article, or claim that may be made by its manufacturer, is not guaranteed or endorsed by the publisher.
